# Patient-derived cancer models: Valuable platforms for anticancer drug testing

**DOI:** 10.3389/fonc.2022.976065

**Published:** 2022-08-12

**Authors:** Sofia Genta, Bryan Coburn, David W. Cescon, Anna Spreafico

**Affiliations:** ^1^ Division of Medical Oncology and Hematology, Princess Margaret Cancer Centre, University Health Network, University of Toronto, Toronto, ON, Canada; ^2^ Division of Infectious Diseases, Toronto General Hospital, University Health Network, Toronto, ON, Canada

**Keywords:** patient-derived model, PDO, drug-testing, co-clinical trial, PDX, cancer

## Abstract

Molecularly targeted treatments and immunotherapy are cornerstones in oncology, with demonstrated efficacy across different tumor types. Nevertheless, the overwhelming majority metastatic disease is incurable due to the onset of drug resistance. Preclinical models including genetically engineered mouse models, patient-derived xenografts and two- and three-dimensional cell cultures have emerged as a useful resource to study mechanisms of cancer progression and predict efficacy of anticancer drugs. However, variables including tumor heterogeneity and the complexities of the microenvironment can impair the faithfulness of these platforms. Here, we will discuss advantages and limitations of these preclinical models, their applicability for drug testing and in co-clinical trials and potential strategies to increase their reliability in predicting responsiveness to anticancer medications.

## 1 Introduction

**Figure 1:**
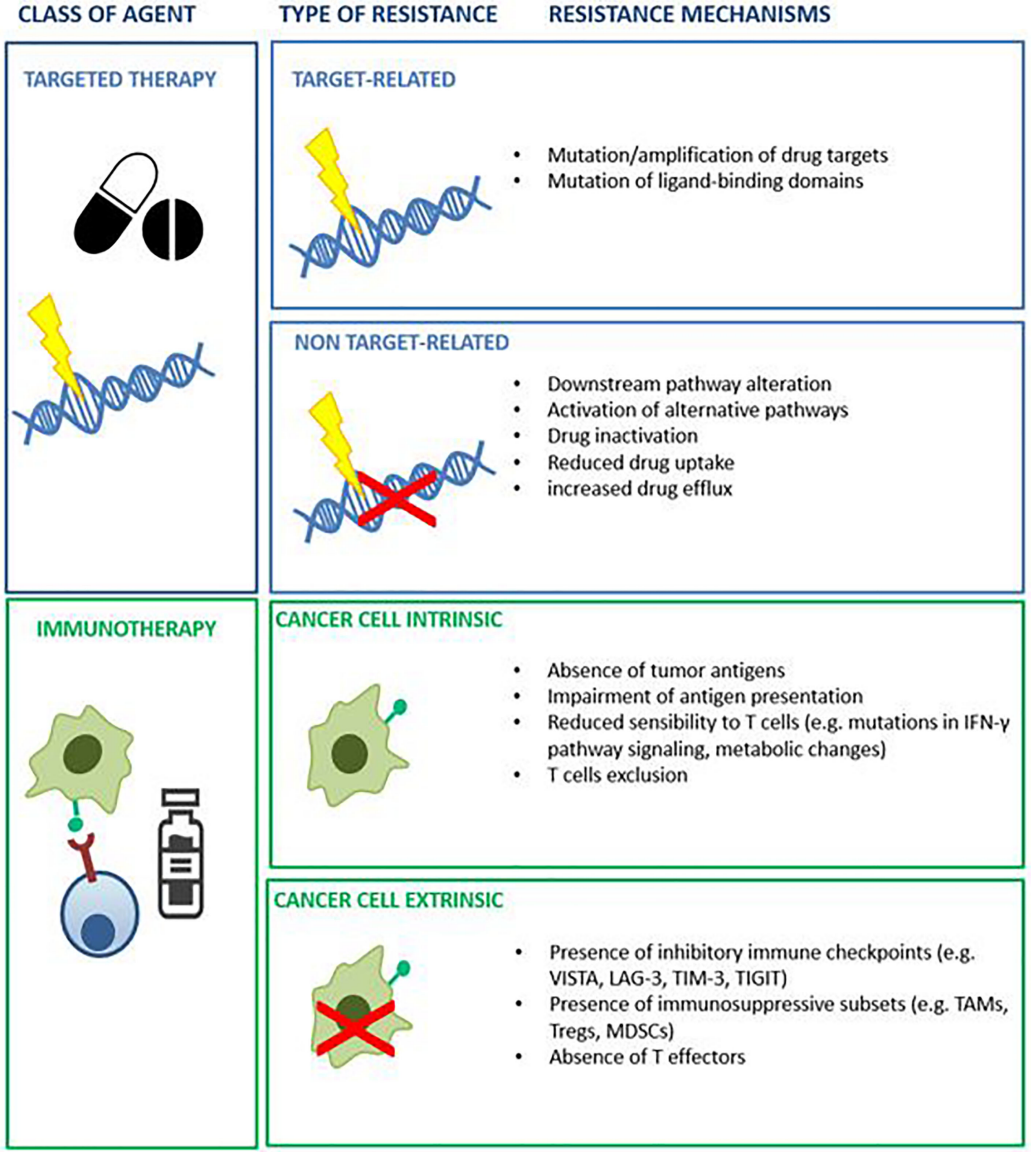
Example of known mechanism of resistance to targeted agents and immunotherapy.

**Figure 2:**
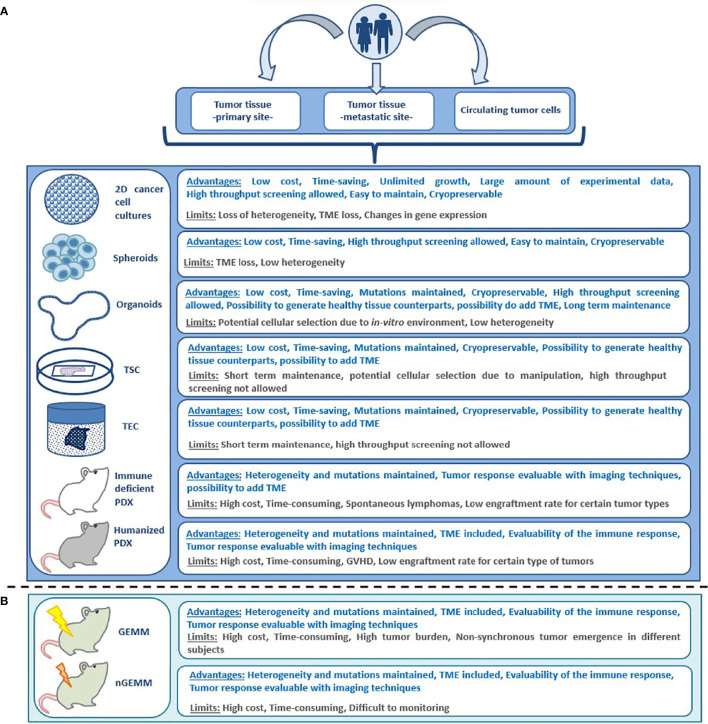
Advantages and limitations of different preclinical models for anticancer drug testing; **(A)** patient derived models, **(B)** non patient-derived animal models. GEMM, genetically engineered mouse model; GVHD, graft versus host disease; nGEMM, non-germinal genetically engineered mouse model; PDX, patient-derived xenograft; TEC, tumor explant culture; TME, tumor microenvironment; 2D, bi-dimensional; TSC, tumor slice culture.

Cancer is a genetic disease that results in cumulative alterations of molecular pathways involved in cell growth, survival and proliferation (1, 2). Until a few decades ago, chemotherapy and endocrine therapy represented the only treatment options for patients with advanced malignancies, and tumor histology was the only benchmark for drug selection (1). The identification of disrupted molecular pathways has notably broadened the therapeutic opportunities for cancer patients, allowing the development of small molecules and monoclonal antibodies exploiting oncogenic driver alterations as drug targets (3). The favorable therapeutic index demonstrated by several of these agents enabled their integration into clinical practice. A large number of such agents are now in clinical use, including epidermal growth factor receptor (*EGFR*) inhibitors in *EGFR* mutated non-small cell lung cancer (NSCLC) (4), anti-epidermal growth factor receptor 2 (*HER2*) agents in HER2-positive breast (5) and gastric cancer (6), B-rapidly accelerated fibro sarcoma (*BRAF*) inhibitors for the treatment of melanoma and other *BRAF* mutated tumors (7). The advent of immunotherapy has ushered in therapeutic strategies that promote immune response against neoplastic cells (8). Many different types of immune-therapeutics have now entered the clinic and some of them, such as immune checkpoint inhibitors (ICIs) and chimeric antigen receptor (CAR)-T cells, have improved patient outcomes (9, 10). In certain settings, including Hodgkin’s lymphoma, melanoma, NSCLC, head and neck, urothelial and renal cell carcinoma, ICIs have replaced previous standard therapies due to overall survival (OS) benefits. Despite the achieved improvement in patient outcomes with the introduction of targeted drugs and immunotherapy, the majority of subjects do not respond to these treatments or experience only a temporary benefit (11, 12). Primary (or intrinsic) and secondary (or acquired) resistance, led by resistance-driving factors in neoplastic tissue before the exposure to an anticancer agent or as a consequence of the antitumor treatment respectively, represent the main reasons for treatment failure with these agents (12). To distinguish between primary and acquired resistance is not always straightforward. Different subpopulations of cancer cells, characterized by specific genomic profiles, usually coexist in the same patient. The phenomenon of intra-patient tumor heterogeneity can be spatial (e.g. in different locations) or temporal (e.g. between primary tumor versus metastasis) (13). Tumor heterogeneity adds complexity to the identification of pre-existing or exposure-induced resistant clones. Multiple mechanisms can be responsible for primary and acquired resistance to specific compounds in different tumor types and their identification is a crucial step in the identification of effective, individualized treatments (14) (**Figure 1**). Patient-derived human cancer models have the potential to retain their distinctive molecular hallmarks, representing a unique opportunity to study cancer cell survival and resistance mechanisms (15–17). If combined with clinical studies, these tools might increase the success of experimental treatments (18, 19). This review will discuss the available preclinical models and the reliability of such platforms to predict the responsiveness to anticancer agents, focusing on patient-derived models (**Figure 2**).

## 2 Two- and three-dimensional cell cultures modeling

### 2.1 Cancer cell lines

Human cancer cell lines represent the earliest and most widely used preclinical model for the investigation of tumor biology and antitumor drugs testing (20). Starting from the 1950s *in vitro* cultures of immortalized cancer cells have been developed from a wide variety of haematological and solid malignancies. These models have been used to assess the effectiveness of investigational anticancer compounds taking advantage of their ease of maintenance and propagation, relatively low cost, reproducibility and high-throughput evaluation (21). However, antitumor activity demonstrated with this approach is often not confirmed in clinical settings, mainly due to the low resemblance to human cancers *in vivo*, and lack of well-defined parameters to translate *in vitro* sensitivity into predicted clinical success (22). This divergence depends on several factors. Firstly, the *in vitro* growth process results in the selection of clones with specific features promoting their survival, outliving other subpopulations. Secondly, the progressive adaptation to culture conditions results in a loss of heterogeneity and differentiation (23). Thirdly, the absence of a natural tumor microenvironment (TME) impairs the evaluation of drugs whose mechanism of action is based on cell-cell interactions or is related to angiogenesis (24). To create platforms with a higher similarity to human cancers and to represent a broader range of tumor types, three-dimensional cultures and *in vivo* models have been developed.

### 2.2 Spheroids

Tumor-derived spheroids are self-assembled micro-aggregates of cancer cells grown in a culture medium, under low-adhesion conditions. They can be generated from cancer cell cultures, patient-derived tumor cells (tumor spheres) or from suspension of single cells from cancer cell lines (25, 26). Spheroid generation is characterized by an initial phase of exponential growth followed by a period of structural organization, which leads to the formation of an external coat of proliferating cells surrounding a necrotic core (27, 28). The cell population located in the inner, hypoxic layers displays a quiescent status. This results in resistance to anticancer drugs exploiting high proliferative rate as a target, mimicking tumor behavior *in vivo* (27). The source of cells used to establish the spheroids has a big impact on the model’s characteristics: for example, spheroids derived from bi-dimensional cell lines will maintain cellular clonality while tumorspheres will display a higher heterogeneity (27, 29).

### 2.3 Organoids

Patient-derived organoids (PDOs) are preclinical models generated from cancer tissue, mechanically or enzymatically dissociated, and then embedded in an extracellular matrix. They differ from spheroids because they are self-organized in three-dimensional structures resembling the architecture and genomic features of the original tissue and retain the capability to regenerate (26, 30). The time required to generate organoids is variable and tumor dependant (31). PDOs of a wide range of malignancies have been established, with success rates up to 80% depending on tumor types (32–40). Mutagenesis technologies such as clustered, regularly interspaced, short palindromic repeats (CRISPR)/CRISPR-associated protein (Cas9) have been used to induce cancer-driving mutations to develop tumoral organoids starting from healthy human tissue (31, 41); an approach particularly useful to study carcinogenesis. Organoids derived from both neoplastic and healthy tissues can be established from the same patient to facilitate the identification of therapeutic agents with high antitumor activity and low impact on physiological tissues (42).

### 2.4 *Ex-vivo* models: Organotypic tumor slice and tumor explant cultures

The term *ex-vivo* is referred to models generated by tissue samples collected from an individual and then preserved in an artificial environment, outside the original organism (43). Organotypic tumor slice cultures (TSCs) are obtained by incubation of thin slices of tissue in controlled conditions allowing oxygen and nutrient distribution (44). This approach has been used to develop models of different types of malignancies, including breast, gastric, head and neck and pancreatic cancer (45–51). When compared to other preclinical models, TSCs offer some advantages including the preservation of an intact tumour microenvironment and a quicker set up, allowing timely drug testing (47). TSCs however, have main limitations such as rapid deterioration of cell viability and tissue architecture as well as unfeasibility of culture propagation (52). Moreover, the resemblance of these models to original tissue is highly influenced by procedural manipulation and sample processing (52). Tumour slice cultures represent only one of the multiple approaches attempted for the generation of *ex-vivo* models. Explanted cancer tissue can be preserved by submerging it in culture media or using a support to keep it in contact with the media such as gelatine sponges (53), grids or culture wells coated by a matrix (54, 55). One of the main advantages of these techniques as compared to tumor slice cultures is that they minimize tissue manipulation and assure higher tissue integrity. On the other hand, tissue slice models may allow a better distribution of anticancer drugs for testing. As for tumor slice cultures, short tissue viability is one of the main limitations for all tumor explant platforms (56). Several attempts have been made in order to delay models deterioration for example through the integration of microfluidic systems allowing prolonged tissue viablity (56).

## 3 *In vivo* models

### 3.1 Non-patient-derived *in vivo* models: The role of engineered mouse models

*In vivo* models enable the evaluation of cancer biology and treatment strategies in a complex organism. Genetically engineered mouse models (GEMMs) are transgenic mice harbouring alleles which lead to the spontaneous development of malignancies in immunocompetent animals (57). The development of GEMMs represented an important milestone in cancer research, as these models have been used to demonstrate that oncogene expression and tumor suppressor gene loss can induce neoplastic transformation of normal cells (58, 59). GEMMs have several limitations. The presence of pathogenic mutations affecting many target cells at an organism or tissue level can result in simultaneous development of multiple malignancies and consequently early death of the model. Furthermore, if present in germline cells, these mutations can affect embryonic viability, cause developmental abnormalities or impair normal tissue development (60). On the other hand, cancer onset in GEMMs can be delayed due to incomplete penetrance of the mutations, resulting in non-synchronous tumor occurrence in different mice and thus impairing the simultaneous evaluation of multiple anticancer agents. The development of non-germline GEMMs and conditional GEMMs, together with novel technologies for genome editing including CRISPR-Cas9 have helped overcome these limitations, enhancing the reliability of engineered mouse models in predicting drug responsiveness (59, 61, 62).

### 3.2 Patient-derived *in-vivo* models

Patient Derived Xenograft (PDXs) are preclinical models established from human neoplastic cells injection or tumor tissue implantation in immune-deficient animal hosts. PDXs are characterized by the maintenance of molecular and cellular heterogeneity of the primary tumor (63). The success rate of PDX establishment depends on multiple variables including the animal recipient, cancer type, and the technique used to implant the tumor (64). Metastatic tumors showing aggressive behaviour more frequently result in successful engraftment. Specific tumor types, such as colorectal or gastric cancer demonstrate a higher probability of engraftment compared with malignancies originating from other sites such as breast (particularly hormone dependent) or kidney (64–66). Many techniques have been used to optimize engraftment, including orthotopic transplant or, in the case of hormone-dependant cancers, the addition of human hormones (53, 67). Furthermore, the probability of obtaining successful engraftment increases with the degree of immunosuppression in the animal host. A greater rate of success can be achieved using animal models lacking functions of both B and T lymphocytes and of natural killer (NK) cells such as non-obese diabetic (NOD)/severe combined immunodeficient (SCID), in particular NOD/SCID/IL-2 receptor-γ deficient (NOG and NSG) and NOD/SCID/Janus kinase 3 deficient (NOJ) mouse models (64). Mice represent the most common type of host used for PDX generation however, other species can be used for this purpose (68–73). *In-vivo* models can also be generated in non-mammalian species, such as zebrafish (68). Both transgenic and xenograft models have been established for different tumor types including endocrine pancreatic cancer (69), multiple myeloma (74), head and neck squamous cell cancer (75), sarcoma (76)and melanoma (70–72), demonstrating some advantages in comparison with traditional mouse models. These include a higher rate of breeding, lower costs of maintenance and the possibility to track malignant cells with fluorescent labelling in the transparent casper zebrafish strain. Moreover, the process of engraftment for zebrafish PDXs is easier and faster compared to their murine counterparts (72, 73). To better recapitulate the original TME, humanized animal models have been developed (77, 78). These models can be obtained by xenotransplanting human immune cells or by engineering the host to express specific human genes. Humanized *in vivo* models, hosting not only human cancer cells but also a human-like TME are particularly suitable to test different anticancer strategies, including immunotherapy.

## 4 Key features of valuable platforms for antitumor drug testing

### 4.1 Genomic and transcriptomic fidelity

Faithful recapitulation of the molecular profile of the original tumor is one of the key characteristics to predict responsiveness to antitumor compounds. PDXs have been considered the most reliable reproduction of human cancers for a long time, retaining more than 80% of the genomic alterations harboured by the engrafted neoplastic tissue (79–81). However, several potential discrepancies have been identified. PDXs often demonstrate a higher aggressiveness with increased proliferation rate -especially at later passages- than human tumors *in situ*. Moreover, some evidence suggests that these models may acquire or select for copy number alterations and single nucleotide variants, or exhibit transcriptional alterations, which can affect the anticancer drug sensitivity (82, 83). Organoids and spheroids have emerged as cost-effective alternatives to animal models, with high genomic concordance with primary tumors (81, 84–88). Multiple factors however, can reduce their resemblance to original cancer tissue or affect their long-term preservation. The purity and the viability of the cancer cells selected to initiate the culture are of crucial importance to ensure successful generation of the model (42). The composition of the culture medium is another key factor. Appropriate nutrient and growth factor modulation are needed to avoid overgrowth of normal cells that would counteract the development of the cancer models (41). The composition of the medium has also demonstrated to influence epigenomic modulation and gene expression (89, 90) and to affect the consumption of glutamine, alanine and other elements from cancer cells (89). *In vitro* culture itself can lead to significant transcriptomic changes, resulting in the upregulation of several growth and metabolism-related pathways such as PI3K, glycolysis and oxidative phosphorylation (90). Some attempts have been made to compare the transcriptional faithfulness of different types of models. As an example, Da Peng et al. have developed the CancerCellNet (CCN), a computational tool evaluating the transcriptomic fidelity of cancer cell lines, PDXs, GEMMs and 3D cultures, through comparison with The Cancer Genome Atlas dataset (91). This approach is burdened by significant limitations, including the small number of models for specific tumor types, the lack of proteomic and epigenomic information, and the fact that the transcriptomic profiles are compared with bulk RNA that includes also non-cancer cells. However, CCN provides some interesting insights indicating that the suitability of different models in proving a faithful reproduction of the original tissue may vary across tumors originating from different sites. This should be considered in studies where generating preclinical models across different tumor types is planned. The application of machine learning algorithms to large datasets collecting genomic and transcriptomic profiles from thousands of patient-derived models can partially overcome the discrepancy with original tumours and optimize drug testing in preclinical studies (92–95). This approach has indeed facilitated the identification of genomic signatures predicting drug sensitivity with a greater precision than single-gene biomarkers (96). Moreover, integrating tumor profiling with analysis of other components of TME, such as the T-cells deep learning models can identify multidimensional biomarker signatures and unveil mechanisms of resistance to anticancer drugs (97, 98).

### 4.2 Preserving tumor heterogeneity

The capability of a model to replicate the molecular features of the original tissue is not enough to ensure success in predicting treatment response. Tumor heterogeneity plays a crucial role in promoting the onset and selection of genomic alterations that lead to cancer cell survival from targeted and immunotherapeutic agents (90). Both PDXs and 3D cultures have been originated from different portions of a same tumor lesion or from different metastatic sites to investigate this phenomenon (99–101). As an example, Li et al. generated PDOs from the primary tumour and paired liver metastases in two patients with colorectal cancer (100). The organoids derived from the metastases demonstrated a more aggressive phenotype with a greater propensity for invasion and higher replication index. Despite providing useful information, this approach is expensive, complex and requires multiple invasive procedures. More importantly, the understanding of genomic aberrations is still limited to specific biopsy sites, hence not practical to predict drug responsiveness. Circulating tumor cells (CTCs), released into the bloodstream from the primary tumour and secondary lesions represent a unique opportunity for the development of complex models for broad evaluation of the genomic landscape of metastatic tumors. CTCs are challenging to isolate as they occur at low frequencies. Data regarding the generation of 3D cultures from CTCs are still limited, however, some successful attempts have been reported (33, 102, 103). In 2014 Gao et al. generated PDOs from CTCs in one patient with prostate cancer with extensive metastatic disease (33). Whole-exome sequencing of PDOs and of a metastatic lymph node resected from the same patient one year before were compared. Only 67% of the point mutations found in the PDOs were identified in the archival tissue. While some of the mutations might be acquired during the culturing process this can reflect tumor heterogeneity. CTCs have also been used to establish animal models, known as cell line derived tumour xenografts (CDXs). Hodgkinson et al. generated CDX models by injecting blood obtained from 4 patients with small cell lung cancer (SCLC), enriched with CTCs (104). Genomic analysis of CDXs shown preservation of original mutational profile, moreover these models mimicked patients responses to chemotherapy. Despite representing a possible strategy to overcome tumor heterogeneity, this approach has some limitations including technical challenges in isolation and expansion of CTCs, lack of stromal and immune components, uncertain representation of different metastatic sites and possible selection of specific clones (105). Despite using CTCs to initiate a patient-derived model the preservation of tumor heterogeneity might be challenged by the fact that cancer cells with particular molecular alterations are more difficult to expand. As an example Li et al. reported a lower rate of success for organoids generation from colorectal cancer with microsatellite instability (MSI) or *BRAF* mutations (106). Interestingly when looking at the possible causes contributing to the failure of these models authors observed that cancer cells harbouring MSI or *BRAF* mutations were more dependant for their survivorship on other components of the tumor microenvironment such as immune cells. This indicate that the maintenance of a proficient TME aside from enabling the evaluation of anticancer therapies such as immunotherapy might be a key factor also for the maintenance of tumor heterogeneity.

### 4.3 Tumor Microenvironment Preservation

#### 4.3.1 Tumor microenvironment preservation in 3D cultures

There has been much recent effort to develop preclinical models with an intact TME preserving functional immune effectors (25, 107). 3D models hosting competent immune cells can be obtained by co-culturing previously expanded immune cells (108). Both spheroid (109–111), and organoid  (112, 113) cultures have been developed with this technique. T cells generated using this process were shown to efficiently kill cancer cells, while they did not show activity against organoids originated from healthy tissue, confirming the maintenance of self-tolerance (112). Courau et al. used this approach to generate immune-competent spheroids from colorectal cancer by co-culturing these models with immune cells obtained from healthy donors (111). They observed rapid infiltration from allogenic transferred T and NK cells, resulting in immune-related cell death. Moreover, they used this platform to test the activity of antibodies targeting natural killer group 2 member D (NKG2D) and its ligands. They observed an enhancement of spheroids immune-mediated killing, driven by an increase of NK infiltration, supporting the utility of this type of model to identify new therapeutic strategies. The co-culturing approach, however, does not fully recapitulate the complexity of the TME, as it lacks native infiltrating immune populations and other key factors, regulating the interaction between cancer and immune system. The extracellular matrix (ECM), the complex of proteins, polysaccharides and other elements surrounding the cells, give structure and sustain both normal and neoplastic tissues. Cancer-associated fibroblasts producing collagen as well as alterations of other elements of ECM, such as hyaluronic acid, metalloproteases and lysyl oxidases are known to promote cancer initiation and invasion through ECM remodeling (114) and have been related to resistance to multiple anticancer agents (115). Moreover, the recognition of the key role played by ECM in cancer resulted in the development of antitumor drugs directly targeting ECM molecules or the cell-matrix crosstalk (115). This led to an urgent need of preclinical models for drug testing retaining a functional ECM. Multiple synthetic and biological materials have been used to develop scaffolds to support the generation of 3D cultures, mimicking ECM. Examples include hydroxyapatite-graphene (116), polyethylene glycol oxide (117), chitosan alginate (118–120), collagen (76, 121) and matrigel (117, 122). Different materials present specific advantages but also limitations. As an example hydrogels are highly biocompatible and recapitulate the biochemical composition of original matrix but offer low mechanical resistance (122). To include original immune populations different 3D models have been developed (123). The feasibility of this approach has been initially demonstrated in healthy human epithelial breast tissue (124). Zumwalde et al. observed the presence of T cells in mammary ductal epithelial PDOs, producing interferon (IFN)-γ and proliferating in response to zoledronic acid. Interestingly, these lymphocytes showed cytotoxic activity towards a triple negative breast carcinoma cell line. The air-liquid interface technique allows to generate PDOs from both healthy and neoplastic tissue with preserved epithelial and mesenchymal components, retaining proficient immune effectors and ECM (123, 125, 126). This method enables organoids to be propagated as epithelial-mesenchymal hybrids using an inner collagen gel–containing transwell with direct air exposure (127). Using this approach Neal et al. generated PDOs from 28 distinct tumor types of human and murine malignancies including colorectal, kidney, lung and pancreatic cancer (123). Human PDO analysis demonstrated the presence of CD3+ tumor infiltrating lymphocytes (TILs), macrophages, B and NK cells. Single-cell gene expression profiling indicated that TILs present within PDOs maintained the original TCR repertoire observed in the tumor biopsy. Protein death 1 (PD-1) expression was observed on the surface of immune cells included in the cultures and the exposure to anti-PD/PD-ligand 1 (PD-L1) agents resulted in the expansion of TILs and in the promotion of neoplastic cells killing. The air-liquid interface technique is not the only possible approach to preserve original TME. Jacob et al. successfully generated PDOs from glioblastoma patients performing microdissection of original tissue into pieces of ≈1 mm diameter instead of dissociation, in order to preserve native cell-cell interactions (128). Single-cell transcriptome analysis shown similar cytokine expression in macrophage and microglia from original tissue and PDOs. Moreover, a similar distribution of cells in PDOs generated at later time points was observed, indicating the capability of this model to preserve and maintain at least in part the features of parental TME. Once established, these models have been used to test multiple antitumor compounds, including CAR-T cells. The resemblance of PDO models to the parental cancer can be further implemented by the organs-on-a-chip technology (129). Through the use of customized microfluidic cell culture devices, this approach allows the vascularization of 3D cultures, mimicking physiological delivery of drugs through the blood vessels (130). Chemo- and biosensors can be integrated in this type of models to optimize the control of oxygen and metabolites levels (131). 3D bioprinting techniques can also be used to develop preclinical models fully recapitulating the architecture of parental tumors, including a functional vascular system and enabling a uniform distribution of different cellular components (132, 133). This approach consists in the controlled deposition of layers of patient-derived cancer and stromal cells, signalling molecules and other biomaterials to generate spheroids or organoids with a functional TME. The use of 3D bioprinting is rapidly expanding and different systems are currently available including extrusion-based bioprinting, laser-based bioprinting, and droplet-based bioprinting (134). 3D bioprinted PDOs and spheroids have already been successful established for multiple cancer types including glioblastoma, neuroblastoma, multiple myeloma, melanoma and cholangiocarcinoma and used to test novel anticancer treatments such as oncolytic viruses (135–141). TEC and TSC represent another possible approach to preserve TME and test immune-oncology strategies, despite the limited time frame for drug-testing. As an example, Sivakumar *et al.* successfully used TSC to test the effect of IFN-γ and PD L1 blockage (47).

#### 4.3.2 Immune-competent *in-vivo* models

The reconstruction of a proficient TME can be applied to *in vivo* models. In GEMMs, spontaneous neoplastic transformation in immunocompetent animals leads to the onset of tumors retaining TME (61, 142). However, the cross-reactivity between the murine and the human targets, especially when the tested agents need antigen presentation by human MHC class I, limits the use of GEMMs to test immunotherapy (143). Humanized animal models are promising platforms for the evaluation of immunotherapeutic strategies. Knock-in mice expressing key human genes regulating the cross-talk between cancer and immune system have been generated, including PD-1 (144), PDL1 (145), LAG3 (145), CTLA4 (77) and IL-15 (146). These platforms could be useful to not only to test drug sensitivity but also to study immune related toxicities associated with monoclonal antibodies specifically binding human targets, such as human PD1 or CTLA4. As an example, Du et al. used CTLA4 humanized mice to test anti-CTLA4 antibodies including ipilimumab, alone and in combination with an anti-PD1 (77). They observed correlation between the development of immune related toxicity and systemic T cell activation with an increased percentage of effector T memory lymphocytes. Humanized PDX models obtained with the engraftment of human immune cells into immunocompromised animals represent another possible approach to obtain animal models suitable for immunotherapy testing (78). This strategy is however burdened by multiple limitations (147). Firstly, the efficacy of the model is limited by the severity of the immunosuppression of the PDX hosts; models with impaired T-cells function but maintained innate immunity will reject human cells. Secondly, since the hematopoietic cells cannot be propagated across multiple animals, this approach requires sequential blood draws from the patients. Finally, the infusion of human hematopoietic cells results in graft versus host disease of PDXs, limiting the timeframe for observation. The transfer of hematopoietic cells is not the only option attempted to generate humanized PDX models. The injection of patient-specific TILs has demonstrated to mimic antitumor responses observed in patients (148). This technique has contributed to identify a dysfunctional subset of CD8+ cells as possible mechanism of resistance to PD 1 inhibitors in NSCLC (149). *Ex-vivo* expansion and subsequent re-infusion of TILs is not the only approach attempted in order to elicit antitumor activity. Yin et al. injected nanoparticle incorporating immunostimulants molecules to induce antitumor activity in endogenous TILs in mouse models. This strategy resulted in CD8+ cells expansion and reduction of regulatory T cells as well as in a delay of tumour growth (150).

### 4.4 Evaluating the impact of microbiome

The human microbiota, the set microorganisms populating human epithelial surfaces, influences the development of several pathologic conditions, including cancer (151). For patient-derived models, this has important conceptual implications: First - for some patient-derived models, the microbial ecology of the model (e.g. the experimental animal’s endogenous microbiota) may influence experimental outcomes and therefore must be accounted for in experimental design and analysis. For example, in murine models, vendor-specific microbiota (152), microbial metabolites (153) and microbiome-tumour neoantigen cross-reactivity (154) have all been implicated in immunotherapy responsiveness. Thus, ignorance of the composition or contribution of the microbiota to model outcomes may result in contradictory findings between investigators, or even within a research group based on variability in the composition of the microbiome between experimental replicates. Secondly - experimental manipulation of the microbiome in patient-derived models may identify novel (host or microbial) targets representing promising therapeutic avenues (155). Both 3D cultures and animal models have been used to mechanistically implicate the microbiome in cancer biology. Using intestinal PDOs, Kadosh et al. observed that the addition of the microbial metabolite gallic acid alters the effect of Trp53 gain of function mutations from tumor-suppressive to pro-oncogenic (156). In adenomatous polyposis coli (*APC*)-mutated mouse models, depletion of *Streptococcus thermophilus* plays a key role in colorectal cancer tumorigenesis (157). The impact of probiotic and high fiber diet on immunotherapy outcomes have been evaluated in several studies showing controversial results (158, 159). Spencer et al. observed an association between higher dietary fiber content and prolonged progression-free survival in melanoma patients receiving ICI while the use of probiotic shown a detrimental effect in the same population (160). To validate these results, they tested the influence of fiber and probiotics on anti-PD1 responses in patient-derived mouse models, confirming that a diet with lower fiber content and addition of a probiotic reduced cytotoxic TILs (160). Finally, mice with microbiome transplanted from human donors (with cancer) represent a type of patient-derived model themselves (161). Routy et al. compared mice with fecal microbiomes transplanted from human patients who responded to anti-PD1 antibody and those receiving fecal transplant from non-responders and found a greater density of intratumoral CD8+ T cells, upregulation of PD L1 and a lower presence of myeloid suppressive cells was in mice transplanted with responders’ stools, suggesting microbiota-induced “hot” TME (162). Critically, these observations have led to the development of novel, microbiome-targeting therapeutic strategies. Shi et al. evaluated the impact of combining a probiotic agent, *Escherichia coli* strain Nissle 1917, to an anti-Transforming Growth Factor, Beta (TGF-β) compound in mice transplanted with breast and hepatocellular carcinoma cells (163). They reported greater tumor growth inhibition and metastasis suppression in models receiving the probiotic. They also observed an increase in the proportion of intratumoral CD8+ T cells and greater numbers of mature dendritic cells in tumour-draining lymph nodes. Fecal transplants from treatment-responsive donors have been investigated in clinical trials assessing their potential to restore sensitivity to immunotherapy in refractory melanoma (164, 165). These data highlight the experimental importance and potential utility of microbiome-informed preclinical studies and the potential for patient-derived microbiome models for the investigation of cancer biology and therapeutic discovery (147).

## 5 Inclusion of patient-derived models in co-clinical trials

Patient-derived platforms have allowed investigators to perform drug testing in models originated from subjects simultaneously receiving therapy in clinical trials. These types of studies, known as co-clinical trials, use laboratory data to guide clinical development or treatment strategies, with the final goal of identifying predictive biomarkers and increasing the rate of success of experimental treatments (166). As an example, Kim et al. tested the fibroblast growth factor receptor (FGFR) inhibitor dovitinib in PDXs derived from squamous cell lung cancer patients treated with the same drug in a clinical study (167). They observed preservation of genomic and histologic profiles of parental tumours in PDXs. The responsiveness to dovitinib displayed by the models recapitulated the clinical outcomes observed in the patients. Gene expression profiling performed in the PDXs indicated upregulation of FGF3 and FGF19 in responders, representing a potential predictive biomarker. The Gustave Roussy MATCH-R project is another example of a co-clinical trial. This is a prospective study aimed at extensive characterization of tumours with acquired resistance to immunotherapeutic agents or targeted therapies. Generation of PDX models for 54 patients with a success rate of 33% and the highest probability of engraftment for urothelial bladder cancer (72.7%) was reported (168). Despite these models being helpful to provide further insights into the mechanism of drug resistance, the authors reported that the outcome data from drug testing were often not timely to guide clinical decision-making. Having a faster turnaround time and being easier to maintain, PDOs might be more suitable for co-clinical studies (169). Yao et al. used PDOs originated from patients with rectal cancer to test the efficacy of chemoradiation and compared it with clinical outcomes (170). The authors reported poor response to chemoradiation in 42/64 patients whose models were resistant, and a good response in 16/17 patients with matched responsive PDOs. They also tested the responsiveness to single components of the chemoradiation regiment (5-FU, irinotecan and radiation) and correlated it with clinical responses. Good clinical outcomes were observed in patients whose PDOs were sensitive to one, two or all the three agents. A good clinical response was reported in 3 patients with PDOs resistant to all three components of the chemoradiation regimen, tested separately. Interestingly, when these models were exposed to the combination, drug synergy was demonstrated in one of the PDOs indicating the potential utility of these models to explore combined treatments to overcome drug resistance. The reliability of PDOs in predicting responses to radiation and chemotherapy in patients with rectal cancer has been evaluated also by further co-clinical studies (171–173). Park et al. developed PDOs from 33 patients radiation for retal adenocarcinoma and confirmed the possibility to use these models to predict sensibility through a machine-learning algorithm (171). In another study, Ganesh et al. were able to generate 65 PDOs from 41 patients with newly diagnosed, metastatic or recurrent rectal cancer with a success rate of 77% (172). Interestingly, 43/65 PDOs were established from samples obtained after exposure to 1 or 2 lines of systemic therapy demonstrating the possibility to generate 3D cultures from pre-treated tumors. Hu et al. have shown that is possible to minimize the time for anticancer drug testing by using microwell arrays that enable to evaluate PDOs sensitivity to hundreds of different compounds at passage 0 (174). Aside from allowing a timely determination of anticancer activity the possibility to perform drug testing at an early stage might reduce the risk of phenotypic changes at later passages and the need of complex culture media with growth factors enrichment. Acoustic droplet printing (ADP) is another approach attempted to decrease the time necessary for PDOs generation (134, 175, 176). This technique enables the development of PDOs in 2 weeks, with preservation of autologous immune cells and integration of a microfluidic system for drug delivery (134). These characteristics make ADP-generated PDOs a promising platform for anticancer drug testing including immunotherapeutic agents. Another critical step to implement co-clinical trial is the definition of fast and standardized methods for the interpretation of antitumor activity in the pre-clinical models. Usually drug sensitivity is estimated using cell viability assays such as ATP-dependent luminescence, tetrazolium-based colorimetric techniques, or fluoresce-based assays (90). These techniques however results PDOs death impairing sequential assessments (177). Emergent technologies such as label-free light microscopy and positron-emission microscopy have been tested to evaluate the antitumor activity of investigational agents in 3D cultures (90, 178, 179). Light microscopy might provide a more precise measurement of antitumor activity because it allows the evaluation of cell viability at single organoid level (90). Moreover, this technique demonstrated the potential to detect not only cytotoxic but also cytostatic activity (90). With the use of positron-emission microscopy was possible to establish that PDOs retain metabolic characteristics of parental tumours, indicating that this technique can be used to monitor responses in the organoid cultures (178). Even when the data obtained from preclinical models are not suitable to guide treatment decisions the information provided by these platforms might be precious to unveil mechanisms underlying drug resistances and develop strategies to restore or increase responsiveness to treatments (180, 181). Moreover, this approach can be used to support the development of non-invasive techniques for the prediction of disease response. As an example, Roy et al. used patient derived PDXs of TNBC to identify [(18)F] fluorodeoxyglucose with positron emission tomography radiomic signatures of response to neoadjuvant chemotherapy (182). A list of co-clinical trials currently ongoing is reported in **Table 1**.

**Table 1:** Ongoing co-clinical trials using patient-derived models for drug testing.

Target population	Intervention	N of patients	Type of preclinical model	NCT number
Stage II-III TNBC	Neoadjuvant chemotherapy base clinical trial not guided by PDX	135	PDX	NCT02124902
Metastatic TNBC	Personalized treatament guided by miniPDX and RNA sequencing	100	miniPDX	NCT04745975
Operated GI cancers	Adjuvant chemotherapy not guided by PDX	120	PDX (zebrafish)	NCT03668418
Pancreatic cancer	Personalized treatment guided by miniPDX	100	miniPDX	NCT04373928
Lung and HNSCC	Standard or experimental systemic treatment not guided by PDX	30	PDX	NCT02597738
Metastatic NSCLC PD-L1+ who failed platinum based treatment	Pembrolizumab not guided by PDX	50	PDX	NCT03134456
Childhood cancers	Personalized treatment guided by molecular profiling and PDX	400	PDX	NCT03336931
Recurrent mantle cell lymphoma	Ibrutinib not guided by PDX	50	PDX	NCT03219047
Localized or metastatic kidney cancer	Personalised treatment guided by PDX	50	PDX	NCT04602702
Metastatic CRPC	Personalised treatment guided by miniPDX	15	miniPDX	NCT03786848
Metastatic CRC	Cetuximab not guided by PDO	80	PDO	NCT04906733
Metastatic pancreatic cancer	Chemotherapy guided by PDO	100	PDO	NCT04931381
Resected pancreatic cancer	Adjuvant chemotherapy guided by PDO	200	PDO	NCT04931394
HNSCC, CRC, breast or epithelial ovarian cancer	Chemotherapy guided by PDO	35	PDO	NCT04279509
Non muscle-invasive bladder cancer	Chemotherapy guided by PDO (instillation)	33	PDO	NCT05024734
Metastatic HER2 negative BC	Chemotherapy guided by PDO	15	PDO	NCT04450706
Operable HER2 positive BC	Chemotherapy + anti-HER2 agents not guided by PDO	94	PDO	NCT04281641
NSCLC	Treatment guided by PDO	100	PDO	NCT04826913
Localized and metastatic CRC	Standard chemotherapy not guided by PDO	120	3D bioprinted PDO	NCT04755907
Advanced BC	Standard therapy not guided by PDO	15	PDO	NCT04655573
Solid tumors	Engineering TCR-T cells	30	PDO	NCT03778814
Locally advanced resectable esophagogastric carcinoma	Standard chemotherapy not guided by PDO	40	PDO	NCT03429816
Locally advanced esophageal cancer	Chemoradiation not guided by PDO	140	PDO	NCT03081988

BC, breast cancer; CRC, colorectal cancer; CRPC, castration-resistant prostate cancer; GI, gastrointestinal; HNSCC, head and neck squamous cell cancer; NSCLC, non-small cell lung cancer; PDO, patient-derived organoids; PDX, patient-derived xenograft; TNBC, triple negative breast cancer.

## 6 Conclusion and future perspectives

Patient-derived models are powerful tools with multiple applications in oncology. Their molecular characterization and incorporation in multiomic biomarker-driven studies is crucial to identify mechanisms of resistance to anticancer treatments and guide the development of effective therapeutic strategies. The Immune Resistance Interrogation Study (IRIS, NCT04243720) currently ongoing at our institution, NEO-R (NCT04504747) and PITCHER (NCT04714957) are some examples of prospective trials using this approach. High genomic and transcriptomic fidelity, preservation of tumor heterogeneity and presence of a proficient TME are some of the key factors that should be implemented to obtain results that can be translated into clinic. The availability of large PDO and PDX repositories combined with the development of machine learning techniques can partially bridge the molecular gap with original tumors and optimize drug testing in preclinical studies (92–95). Until today, tumor heterogeneity represented a main pitfall, jeopardizing the reliability of preclinical models in predicting drug sensitivity. Although there is a big caveat on the success and expansion of CTCs, there is a question of whether they could help overcome this limitation by capturing inter- and intratumoral heterogeneity. The use of 3D bioprinting techniques might enable the development of complex 3D cultures starting from CTCs, comprising proficient autologous immune cells and vascular system. Aside from overcoming tumour heterogeneity, the use of CTCs as primary source for the development of cancer models may offer further advantages. Due to its relatively low invasiveness, a liquid biopsy-based approach might be particularly useful to investigate mechanisms of acquired resistance through the comparison of CDXs obtained by sequential blood draws. Finally multiple strategies have been implemented to preserve a functional TME leading to the development of complex *in-vivo*, *ex-vivo* and *in-vitro* models. These platforms may enable a deeper understanding of the factors regulating the networking between cancer cells and immune system, such as microbiome and ECM. Moreover humanized *in-vivo* models and 3D cultures retaining functional immune effectors and ECM are promising tools to test not only immunotherapy but also novel therapeutic strategies targeting critical processes underlying cancer initiation and progression such as matrix deposition and remodeling. Further elements should be taken into consideration if the patient derived model is intended to guide treatment selection in the context of a co-clinical trial. The models are highly time-sensitive. Despite the molecular affinity with the parental tumors, mouse PDXs need months to be established; therefore, limiting their applicability in treatment decision making, as demonstrated by the MATCH-R study (168). Alternative models including 3D cell cultures, along with innovative techniques such as 3D bioprinting and microwell arrays could overcome these limitations and have higher molecular fidelity thanks to the limited number of passages between tissue collection and drug-testing.

## Author contributions

SG: conceptualization and original draft preparation**;** BC, DC, and AS: supervision and manuscript review/editing. All authors have read and agreed to the published version of the manuscript.

## Conflict of interest

BC: Research funding: Nubiyota, Sanofi. DC: Consulting/Advisory: AstraZeneca, Extract Sciences, Eisai, Gilead, GlaxoSmithKline, Inivata, Merck, Novartis, Pfizer and Roche. Research funding to institution: AstraZeneca, Gilead, GlaxoSmithKline, Inivata, Merck, Pfizer and Roche. Patent (US62/675,228) for methods of treating cancers characterized by a high expression level of spindle and kinetochore associated complex subunit 3 (ska3) gene. AS: Consultant for (Advisory Board): Merck (compensated), Bristol-Myers Squibb (compensated), Novartis (compensated), Oncorus (compensated), Janssen (compensated). Grant/Research support from (Clinical Trials): Novartis, Bristol-Myers Squibb, Symphogen AstraZeneca/Medimmune, Merck, Surface Oncology, Northern Biologics, Janssen Oncology/Johnson & Johnson, Roche, Regeneron, Alkermes, Array Biopharma/Pfizer, GSK.

The remaining author declares that the research was conducted in the absence of any commercial or financial relationships that could be construed as a potential conflict of interest.

## Publisher’s note

All claims expressed in this article are solely those of the authors and do not necessarily represent those of their affiliated organizations, or those of the publisher, the editors and the reviewers. Any product that may be evaluated in this article, or claim that may be made by its manufacturer, is not guaranteed or endorsed by the publisher.

## Abbreviations

ADP, Acoustic droplet printing; BC, breast cancer; BRAF, B-rapidly accelerated fibro sarcoma; CAR-T, chimeric antigen receptor T-cell; CCN, CancerCellNet; CDX, cell line derived tumor xenograft; CRC, colorectal cancer; CRISPR-Cas9, clustered regularly interspaced short palindromic repeats/associated protein; CTC, circulating tumor cell; GEMM, Genetically engineered mouse model; ECM, extracellular matrix; EGFR, epidermal growth factor receptor; HER-2, epidermal growth factor receptor 2; HNSCC, head and neck squamous cell cancer; ICI, immune checkpoint inhibitors; IFN-γ, interferon gamma; MHC, major histocompatibility complex; NSCLC, non-small cell lung cancer; NK, natural killer; NOD/SCID, non-obese diabetic/severe combined immunodeficient; OS, overall survival; PD-1, protein death 1; PD-L1, protein death 1ligand; PDO, patient-derived organoid; PDX, patient derived xenograft; TCR, T cell receptor; TEC, tumour explant cultures; TIL, tumor infiltrating lymphocyte; TME, tumor microenvronment; TNBC, triple negative breast cancer; TSC, tumour slice cultures.
